# Personal protective equipment utilization and its association with educational status among industry workers in Ethiopia: A systematic review and meta-analysis protocol

**DOI:** 10.1371/journal.pone.0299957

**Published:** 2024-04-18

**Authors:** Sisay Abebe Debela, Yonatal Mesfin Tefera, Mulualem Endeshaw, Chala Daba, Endashaw Abebe Debela, Amana Ogeto Luke, Teferi Atomsa, Solomon Seyoum, Mesfin Gebrehiwot

**Affiliations:** 1 Department of Public Health, College of Medicine and Health Sciences, Salale University, Fitche, Ethiopia; 2 Adelaide Exposure Science and Health, School of Public Health, University of Adelaide, Adelaide, Australia; 3 Department of Public Health, Rift Valley University, Jimma, Ethiopia; 4 Department of Environmental Health, College of Medicine and Health Sciences, Wollo University, Dessie, Ethiopia; 5 School of Medicine, Adama Hospital and Medical College, Adama, Ethiopia; 6 Department of Anatomy, St. Paul’s Hospital Millennium Medical College, Addis Ababa, Ethiopia; Haile Selassie I Hospital: Yekatit 12 Hospital Medical College, ETHIOPIA

## Abstract

**Introduction:**

Ethiopia is experiencing high prevalence of occupational morbidity and disability. One of the main contributing reasons is a low utilization of personal protective equipment (PPE). Previous studies on PPE utilization and association with educational status among industry workers were largely inconsistent. Therefore, this meta-analysis is aimed to pool the magnitude of PPE utilization and its association with educational status among industry workers in Ethiopia.

**Method:**

A compressive search of international databases and libraries including Scopus, PubMed, MedNar, Embase, MEDLINE, the web of science, Google Scholar, the JBI Library, African Journals Online, and Science direct will be carried out to locate published reports. Two independent reviewers will screen the records for inclusion using standardized JBI tools. Before extracting and synthesizing data, the selected studies will undergo a rigorous critical appraisal. If appropriate, a meta-analysis will be conducted. Cochrane Q-test and I^2^-test statistics will be used to assess the heterogeneity between studies. If necessary, meta-regression and subgroup analyses will be conducted to explore potential reasons for any inconsistency and heterogeneity. Sensitivity analysis will be performed to assess the effect of a single study on the pooled magnitude estimates. Funnel plots, along with Egger’s and Begg’s tests, will be used to assess the presence of publication bias.

**PROSPERO registration number:** PROSPERO, CRD42022364562

## Introduction

The hierarchy of workplace hazard control is a step-by-step framework that prioritizes controls in a specific order. It begins with higher-level measures, such as elimination (complete hazard removal), substitution (replacing hazards with safer alternatives), engineering controls (modifying the environment to protect workers), and administrative controls (establishing safety protocols and training). These measures are designed to eliminate, reduce, or manage hazards at their source. Personal protective equipment (PPE) is considered a last resort when higher-level controls are impractical or insufficient [1]. Personal protective equipment (PPE) are special gears or apparatus designed to safeguard the user against potential occupational health risks. It comprises equipment, such as safety helmets, hard hats, face masks, gloves, eye protection, boots, ear plugs, high-visibility clothes, safety footwear, and safety harnesses. PPE utilization is essential for preventing occupational injuries and illnesses caused by workplace risk factors and stressors. Studies show that wearing PPE at all times could help reduce workplace accidents [2, 3], and could contribute to the national economy through increased productivity, job motivation, product quality, job satisfaction, and overall worker and societal quality. However, the utilization of PPE by employees is affected by socioeconomic status, behavioral, and workplace factors [4].

Unfortunately, millions of people in productive age groups work in uncomfortable and risky conditions around the world as a result of inappropriate or inadequate PPE use [5, 6]. It continues to be one of the leading causes of work-related deaths and disabilities [7]. Research findings showed that factory workers could lack adequate information about protective precautions and be less aware of the safety risks resulting from multiple activities in their workplaces [4, 8–10]. Globally, 1.7 billion workers in different service sectors are at high risk of occupational hazards. Failure to use PPE that was available at the time of the accident is reported to be responsible for 34% of occupational accidents [11]. Likewise, improper PPE use could account for up to 13% of occupational hazards [12]. According to Takala et al, the leading causes of death associated with improper utilization of PPE in the workplaces of developing countries are cancer (32%), circulatory disorders (23%), communicable diseases (17%), and occupational accidents (18%) [7]. According to a study by Baye et al, using PPE and following safety protocols can prevent more than 90% of workplace injuries [2].

Ethiopia is a member of the International Labor Organization (ILO) and accepted conventions governing the health and safety of factory workers in 1923 [6]. ILO signatory nations ratified Convention No. 155/1981 on occupational safety and health to have national occupational safety and health policy [9, 13]. Ethiopian Labor Proclamation No. 377/2003 declares the right of labor and detailed employers obligations [14]. Under the proclamation, employers must take precautionary measures ranging from instructing or informing employees about the risks of their specific jobs to providing them with appropriate protective tools and safety equipment. Various organizations in Ethiopia promote employee engagement in occupational health and safety [8]. However, there is no noticeable responsibility for the flow of work, concise collaboration, or direct responsibility for employees safety [9]. This implies that the documents were shelved in the ministry of social and labor affairs. In addition, there is lack of comprehensive national strategy for workplace safety and health for industry workers in the country. As a result, occupational morbidity, disability, and death without the utilization of PPE have become public health concerns [15, 16]. In Ethiopia, only a small percentage of workers, ranging from 5% to 10%, have appropriate occupational health services at their workplaces [17].

Although several studies have been carried out in Ethiopia to investigate the levels of PPE utilization, the results of these studies differ widely among industry workers [18–23]. Some of the findings indicated good progress in the rate of PPE utilization, whereas others highlighted the awkward aspect. Furthermore, educated worker are believed to learn how to effectively utilize PPE to safeguard their health and well-being in the workplace. That is why education and awareness creation are among the 16 packages encompassed in the Ethiopian health extension packages [24]. In spite of all, previous findings concerning the influence of education on PPE utilization remain inconsistent and contradicting [25–27], which underscores the need for further investigation.

The aim of this paper is, therefore, to develop a systematic review and meta-analysis protocol for the estimation of the pooled magnitude of PPE utilization and its relationship with educational level among industry workers in Ethiopia. The final output at the national level will provide a more reliable overall figure both preceding and following the COVID-19 pandemic. It will provide a valuable insight for decision-makers in assessing the country’s stance on PPE utilization and shaping policies related to PPE use in industrial settings. Publishing this review protocol is important for lessening the influence of biases from the authors, enhancing the transparency of methods and processes, minimizing duplication, and enabling peer review of the proposed methods.

### Review questions

1. What is the pooled magnitude of PPE utilization among industry workers in Ethiopia?
2. What is the association between PPE utilization and educational status among industry workers in Ethiopia?

## Methodology

### Study registration

The systematic review and meta-analysis will follow the JBI methodology for systematic reviews of effectiveness [28]. The protocol has been registered in PROSPERO (CRD42022364562).

### Search strategy

All potentially relevant articles, grey literature, and government reports will be meticulously searched to conduct this study. International databases and libraries, such as Scopus, PubMed, the web of science, MEDLINE, Embase, Google Scholar, MedNar, the JBI Library, African Journals Online, and Science direct, will be included to search relevant studies. We will retrieve grey literature using Google Scholar and Google searches. We will also review reference lists of identified studies in order to find and retrieve additional articles. Unpublished studies will be retrieved from the official websites of international and local organizations and universities. An extensive search will be done from the databases using the following keywords: "prevalence", "proportion", "magnitude", "personal protective equipment", "personal", "protective", "equipment", "use", "utilization", "industry", "factory", "workers", "textile industry", "tannery industry", "flour industry", "construction factory", "Fuel station", "cement industry", "cotton ginning industry", "wood industry", "metal industry", "factors", "determinants", "predictors", "factors associated", "associated factors", "risk factors", "Ethiopia" to collect published articles without restring the study period. We will undertake advanced searching by combining the search terms using Boolean operators. The search for all articles will be conducted from 1^st^ to 15^th^ February, 2024.

### Study selection and eligibility criteria

The review will include Ethiopian studies that reported the level of PPE utilization, as well as articles published in scientific journals and grey literature. Only study reports written in English and full-text articles will be considered. Furthermore, the review will look at all observational studies (cohort, cross-sectional, and case-control). Articles that remain inaccessible for full-text retrieval, despite at least three email attempts to reach the primary study’s principal investigators, will be excluded. Two independent reviewers (SAD and CD) will screen the records for inclusion. Each reviewer will carry out the evaluation independently. Disagreements among reviewers will be discussed with the review team members until a consensus is reached.

#### Inclusion criteria

Articles meeting the specified criteria will be incorporated into this systematic review

#### Population

This systematic review and meta-analysis will exclusively include studies conducted among industrial workers in Ethiopia

#### Exposure

Workers in the workplace who use PPE.

#### Comparison

Workers who use PPE and are educated, people who use PPE but are not educated, people who do not use PPE and are educated, and people who do not use PPE and are not educated.

#### Outcome

This study will determine the pooled magnitude of PPE utilization, and its association with educational status among industry workers in Ethiopia as primary and secondary outcomes, respectively. The magnitude will be computed as the proportion of the number of individuals who utilize PPE to the total number of participants multiplied by 100. The studies eligible for inclusion should present at least one of the anticipated outcomes.

#### Study setting

Only studies conducted in Ethiopia will be considered.

#### Study design

All observational studies (including cohort, cross-sectional, and case-control studies) detailing the use of PPE among industrial workers will be included.

#### Publication

Both published and unpublished studies will be included.

#### Exclusion criteria

Studies with no full text, published in languages other than English, unidentified reports, communications, editorials, case reports, letters to the editor, case series, and qualitative studies will be excluded.

### Quality assessment and data extraction

**Fig 1:**
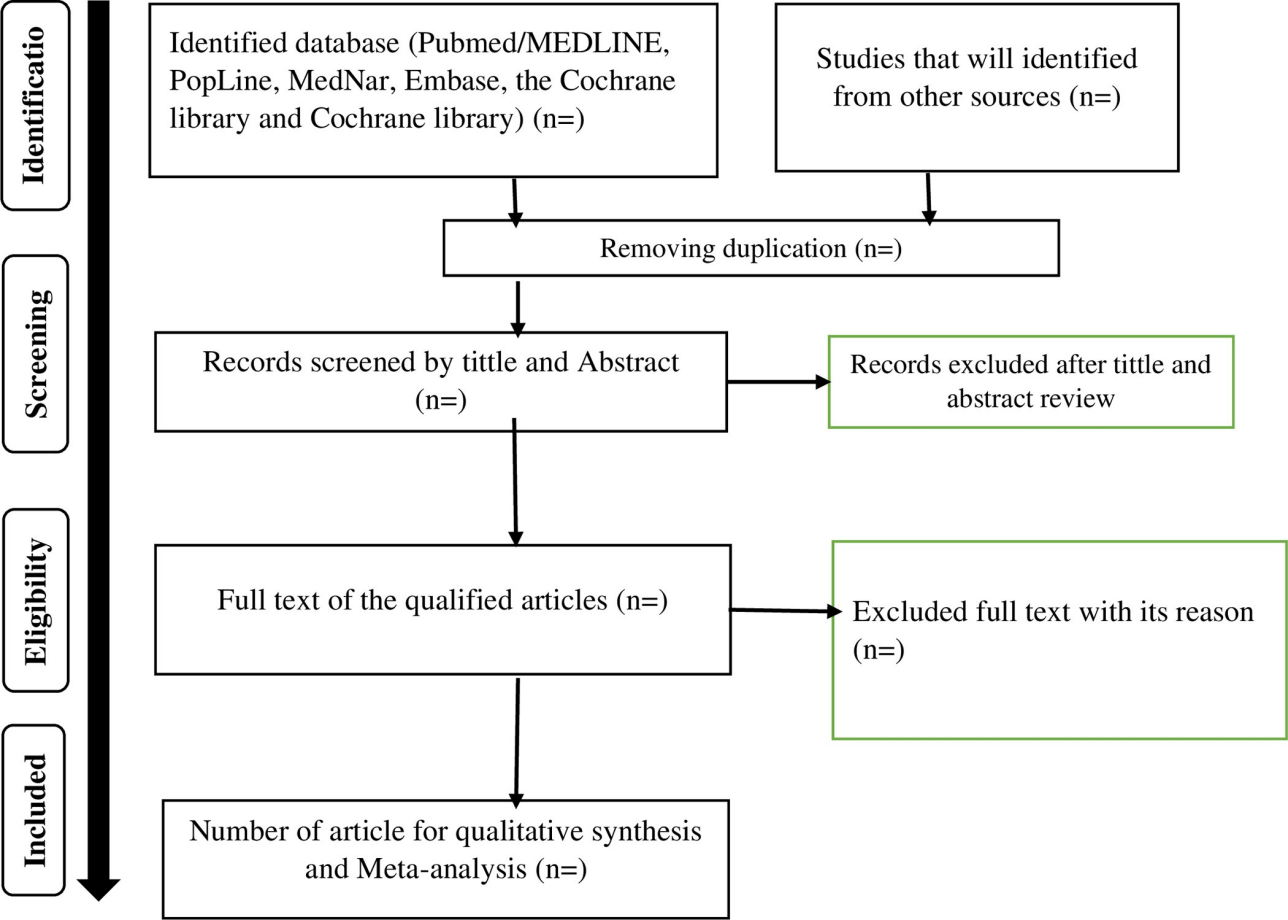
PRISIMA flow diagram of studies to be identified.

Duplicate records from all database search results will be removed using reference management software (EndNote X7). Four reviewers (SAD, CD, EAD, and YMT) will assess the titles and abstracts, and exclude records that do not meet our criteria. The Joanna Briggs Institute Meta-Analysis of Statistics Assessment and Review Instrument (JBI-MAStARI) will be employed for the objective assessment of papers [29]. A standardized data extraction format will be used by two independent reviewers to extract the data (SAD and CD). The data extraction spread sheet will include primary author name, year of publication, study period (before and after the COVID 19), study setting, type of industry, study design, sample size, study period, number of sample size, response rate, PPE use (Yes/No), educational status (educated/ no formal education), quality of the paper result, people who are using PPE and educated, people who are using PPE and are not educated, people who are not using PPE and educated, and people who are not using PPE and are not educated. When there is disagreement between reviewers, it will be discussed with the other members of the review team until an agreement is reached. A third reviewer will be called upon to address any discrepancies between the two independent reviewers. PRISMA flow diagram will be used to show the review process of the studies (Fig 1).

### Assessing certainty in the findings

The Grading of Recommendations, Assessment, Development, and Evaluation (GRADE) approach will be used to evaluate the quality of evidence and create a summary of findings. For this, the five domains established by GRADE guidelines (risk of bias, inconsistency, indirectness, imprecision, and publication bias) will be used. The outcomes reported in the summary of findings will include the pooled magnitude of PPE utilization and its association with educational status.

### Data analysis and synthesis

Using the ‘generate’ command in STATA 16.0, from the original research, odds ratios (OR) and standard errors will be created for all included studies. The p-values of the Cochrane Q-test and I^2^-test statistics will be used to assess the heterogeneity among studies. A univariate meta-regression will be used to assess the causes of heterogeneity across each research study when the results of a meta-analysis show significant heterogeneity. Publication bias will be visually assessed through a funnel plot. Asymmetry in the funnel plot suggests potential publication bias. Additionally, Egger’s test will be performed to identify significant publication bias, with a p-value below 0.10 indicating its presence [30]. Because Egger’s test is more specific than Begg’s test, it will be used to assess publication bias. We will use the log odds ratio to determine the association between workers’ educational status and their PPE utilization. Furthermore, sensitivity analysis using a random effects model will be performed to assess the effect of a single study on the pooled magnitude estimates [31]. Sub-group analysis will be used to lower the random variations between the primary study’s inter-group estimates, and the analysis will be done based on study settings (i.e., region). A univariable meta-regression analysis will also be conducted using the year of publication and the outcome variable. Stata software (Version 16.0) will be used for all data manipulation and statistical analysis. Where meta-analysis or statistical pooling is not possible, the findings will be presented in narrative form, with tables and figures to help with data presentation.

### Operational definitions

#### PPE Utilization

The participant who used PPE (Yes or No).

#### Educational categories

The primary studies classified education for the study participant as 1) not attended formal education, 2) attended primary education (grades 1–8), 3) attended secondary Educations, and 4) attended college and above.

## Discussion

Numerous studies conducted in Ethiopia have sought to determine the extent of PPE use among industry workers. Nevertheless, the results of these studies have been debated and inconclusive. The compilation and synthesis of evidence represent a crucial step toward gaining a clearer understanding of the influence of education on PPE use. In our review and meta-analysis, we will determine the pooled magnitude of PPE usage among industry workers across Ethiopia and investigate the influence of educational status on the use of PPE.

To enhance feasibility, the protocol plan intends to conduct searches without restricting the type of industry, workers, PPE types, the COVID-19 period, educational status, and the frequency and duration of PPE use, thereby minimizing the risk of overlooking relevant studies on PPE use among industry workers. This will also ensure the retrieval of as many studies as possible. Additionally, language restriction is incorporated into this protocol to international libraries and databases. Through our preliminary search on PubMed, we have identified over 26,000 studies concerning PPE use in the industry (S1 File). Therefore, our search strategy, inclusion criteria, and selection of databases are structured to ensure the feasibility of conducting a systematic review.

## Supporting information

S1 Checklist (DOCX)

S1 Data (DOCX)

S1 File (DOCX)
