# Supplementary material for: Personal protective equipment utilization and its association with educational status among industry workers in Ethiopia: A systematic review and meta-analysis protocol
Source: PLoS One. 2024 Apr 18;19(4):e0299957. doi: 10.1371/journal.pone.0299957 (PMC11025741; doi:10.1371/journal.pone.0299957)
Supplement: S1 Data — (DOCX) [file pone.0299957.s002.docx]

| Extractor 1 |  |
| --- | --- |
| Extractor 2 |  |
| Citation Details |  |
| ID |  |
| Authors |  |
| Tittle |  |
| Year of Publication |  |
| DOI link |  |
| Study Type |  |
| Region |  |
| Study setting |  |
| Type of Industry |  |
| Study period |  |
| Sample size |  |
| Response rate |  |
| PPE Use |  |
| Not using PPE |  |
| No attend formal education |  |
| Attend formal education |  |
| People who are using Personal protective equipment and Educated |  |
| People who are using Personal protective equipment and are not Educated |  |
| People who are not using Personal protective equipment and Educated |  |
| People who are not using Personal protective equipment and are not Educated |  |
