# Supplementary material for: Personal protective equipment utilization and its association with educational status among industry workers in Ethiopia: A systematic review and meta-analysis protocol
Source: PLoS One. 2024 Apr 18;19(4):e0299957. doi: 10.1371/journal.pone.0299957 (PMC11025741; doi:10.1371/journal.pone.0299957)
Supplement: S1 File — (DOCX) [file pone.0299957.s003.docx]

**PubMed trials**

**Date searched: May 31, 2023**

**Results retrieved: 26716**

Prevalence OR proportion OR magnitude AND personal protective equipment OR PPE OR personal OR safety equipment AND use* OR utilization OR practice AND industry OR factory OR small scale industry OR small scale enterprise AND workers OR small scale industry workers OR large scale industry OR large scale industry workers AND textile industry OR tannery industry OR flour industry OR construction factory OR Fuel station OR cement industry OR cotton ginning industry OR wood industry OR metal industry OR factors OR determinants OR predictors OR factors associated OR associated factors OR risk factors AND Ethiopia"

((((((((((("epidemiology"[MeSH Subheading] OR "epidemiology"[All Fields] OR "prevalence"[All Fields] OR "prevalence"[MeSH Terms] OR "prevalance"[All Fields] OR "prevalences"[All Fields] OR "prevalence s"[All Fields] OR "prevalent"[All Fields] OR "prevalently"[All Fields] OR "prevalents"[All Fields] OR ("proportion"[All Fields] OR "proportions"[All Fields]) OR ("magnitude"[All Fields] OR "magnitudes"[All Fields])) AND ("personal protective equipment"[MeSH Terms] OR ("personal"[All Fields] AND "protective"[All Fields] AND "equipment"[All Fields]) OR "personal protective equipment"[All Fields])) OR ("polit philos econ"[Journal] OR "ppe"[All Fields]) OR ("person s"[All Fields] OR "personable"[All Fields] OR "personableness"[All Fields] OR "personal"[All Fields] OR "personalisation"[All Fields] OR "personalise"[All Fields] OR "personalised"[All Fields] OR "personalising"[All Fields] OR "personality"[MeSH Terms] OR "personality"[All Fields] OR "personalities"[All Fields] OR "personality s"[All Fields] OR "personalization"[All Fields] OR "personalize"[All Fields] OR "personalized"[All Fields] OR "personalizes"[All Fields] OR "personalizing"[All Fields] OR "personally"[All Fields] OR "personals"[All Fields] OR "persons"[MeSH Terms] OR "persons"[All Fields] OR "person"[All Fields]) OR ("protective devices"[MeSH Terms] OR ("protective"[All Fields] AND "devices"[All Fields]) OR "protective devices"[All Fields] OR ("safety"[All Fields] AND "equipment"[All Fields]) OR "safety equipment"[All Fields])) AND "use"[All Fields]) OR ("statistics and numerical data"[MeSH Subheading] OR ("statistics"[All Fields] AND "numerical"[All Fields] AND "data"[All Fields]) OR "statistics and numerical data"[All Fields] OR "utilization"[All Fields] OR "utilisation"[All Fields] OR "utilisations"[All Fields] OR "utilise"[All Fields] OR "utilised"[All Fields] OR "utilises"[All Fields] OR "utilising"[All Fields] OR "utilities"[All Fields] OR "utility"[All Fields] OR "utilizations"[All Fields] OR "utilize"[All Fields] OR "utilized"[All Fields] OR "utilizer"[All Fields] OR "utilizers"[All Fields] OR "utilizes"[All Fields] OR "utilizing"[All Fields]) OR ("practicability"[All Fields] OR "practicable"[All Fields] OR "practical"[All Fields] OR "practicalities"[All Fields] OR "practicality"[All Fields] OR "practically"[All Fields] OR "practicals"[All Fields] OR "practice"[All Fields] OR "practice s"[All Fields] OR "practiced"[All Fields] OR "practices"[All Fields] OR "practicing"[All Fields])) AND ("industrial development"[MeSH Terms] OR ("industrial"[All Fields] AND "development"[All Fields]) OR "industrial development"[All Fields] OR "industrialization"[All Fields] OR "industrialize"[All Fields] OR "industrialized"[All Fields] OR "industrializing"[All Fields] OR "industrially"[All Fields] OR "industrials"[All Fields] OR "industry"[MeSH Terms] OR "industry"[All Fields] OR "industrial"[All Fields] OR "industries"[All Fields] OR "industry s"[All Fields])) OR ("factories"[All Fields] OR "factory"[All Fields] OR "factory s"[All Fields]) OR (("small"[Journal] OR "small"[All Fields]) AND ("scale s"[All Fields] OR "scaled"[All Fields] OR "scaling"[All Fields] OR "scalings"[All Fields] OR "weights and measures"[MeSH Terms] OR ("weights"[All Fields] AND "measures"[All Fields]) OR "weights and measures"[All Fields] OR "scale"[All Fields] OR "scales"[All Fields]) AND ("industrial development"[MeSH Terms] OR ("industrial"[All Fields] AND "development"[All Fields]) OR "industrial development"[All Fields] OR "industrialization"[All Fields] OR "industrialize"[All Fields] OR "industrialized"[All Fields] OR "industrializing"[All Fields] OR "industrially"[All Fields] OR "industrials"[All Fields] OR "industry"[MeSH Terms] OR "industry"[All Fields] OR "industrial"[All Fields] OR "industries"[All Fields] OR "industry s"[All Fields])) OR (("small"[Journal] OR "small"[All Fields]) AND ("scale s"[All Fields] OR "scaled"[All Fields] OR "scaling"[All Fields] OR "scalings"[All Fields] OR "weights and measures"[MeSH Terms] OR ("weights"[All Fields] AND "measures"[All Fields]) OR "weights and measures"[All Fields] OR "scale"[All Fields] OR "scales"[All Fields]) AND ("enterprise"[All Fields] OR "enterprise s"[All Fields] OR "enterprisers"[All Fields] OR "enterprises"[All Fields] OR "enterprising"[All Fields]))) AND ("occupational groups"[MeSH Terms] OR ("occupational"[All Fields] AND "groups"[All Fields]) OR "occupational groups"[All Fields] OR "worker"[All Fields] OR "workers"[All Fields] OR "worker s"[All Fields])) OR (("small"[Journal] OR "small"[All Fields]) AND ("scale s"[All Fields] OR "scaled"[All Fields] OR "scaling"[All Fields] OR "scalings"[All Fields] OR "weights and measures"[MeSH Terms] OR ("weights"[All Fields] AND "measures"[All Fields]) OR "weights and measures"[All Fields] OR "scale"[All Fields] OR "scales"[All Fields]) AND ("industrial development"[MeSH Terms] OR ("industrial"[All Fields] AND "development"[All Fields]) OR "industrial development"[All Fields] OR "industrialization"[All Fields] OR "industrialize"[All Fields] OR "industrialized"[All Fields] OR "industrializing"[All Fields] OR "industrially"[All Fields] OR "industrials"[All Fields] OR "industry"[MeSH Terms] OR "industry"[All Fields] OR "industrial"[All Fields] OR "industries"[All Fields] OR "industry s"[All Fields]) AND ("occupational groups"[MeSH Terms] OR ("occupational"[All Fields] AND "groups"[All Fields]) OR "occupational groups"[All Fields] OR "worker"[All Fields] OR "workers"[All Fields] OR "worker s"[All Fields])) OR (("large"[All Fields] OR "largely"[All Fields] OR "larges"[All Fields]) AND ("scale s"[All Fields] OR "scaled"[All Fields] OR "scaling"[All Fields] OR "scalings"[All Fields] OR "weights and measures"[MeSH Terms] OR ("weights"[All Fields] AND "measures"[All Fields]) OR "weights and measures"[All Fields] OR "scale"[All Fields] OR "scales"[All Fields]) AND ("industrial development"[MeSH Terms] OR ("industrial"[All Fields] AND "development"[All Fields]) OR "industrial development"[All Fields] OR "industrialization"[All Fields] OR "industrialize"[All Fields] OR "industrialized"[All Fields] OR "industrializing"[All Fields] OR "industrially"[All Fields] OR "industrials"[All Fields] OR "industry"[MeSH Terms] OR "industry"[All Fields] OR "industrial"[All Fields] OR "industries"[All Fields] OR "industry s"[All Fields])) OR (("large"[All Fields] OR "largely"[All Fields] OR "larges"[All Fields]) AND ("scale s"[All Fields] OR "scaled"[All Fields] OR "scaling"[All Fields] OR "scalings"[All Fields] OR "weights and measures"[MeSH Terms] OR ("weights"[All Fields] AND "measures"[All Fields]) OR "weights and measures"[All Fields] OR "scale"[All Fields] OR "scales"[All Fields]) AND ("industrial development"[MeSH Terms] OR ("industrial"[All Fields] AND "development"[All Fields]) OR "industrial development"[All Fields] OR "industrialization"[All Fields] OR "industrialize"[All Fields] OR "industrialized"[All Fields] OR "industrializing"[All Fields] OR "industrially"[All Fields] OR "industrials"[All Fields] OR "industry"[MeSH Terms] OR "industry"[All Fields] OR "industrial"[All Fields] OR "industries"[All Fields] OR "industry s"[All Fields]) AND ("occupational groups"[MeSH Terms] OR ("occupational"[All Fields] AND "groups"[All Fields]) OR "occupational groups"[All Fields] OR "worker"[All Fields] OR "workers"[All Fields] OR "worker s"[All Fields]))) AND ("textile industry"[MeSH Terms] OR ("textile"[All Fields] AND "industry"[All Fields]) OR "textile industry"[All Fields])) OR (("tanneries"[All Fields] OR "tannery"[All Fields]) AND ("industrial development"[MeSH Terms] OR ("industrial"[All Fields] AND "development"[All Fields]) OR "industrial development"[All Fields] OR "industrialization"[All Fields] OR "industrialize"[All Fields] OR "industrialized"[All Fields] OR "industrializing"[All Fields] OR "industrially"[All Fields] OR "industrials"[All Fields] OR "industry"[MeSH Terms] OR "industry"[All Fields] OR "industrial"[All Fields] OR "industries"[All Fields] OR "industry s"[All Fields])) OR (("flour"[MeSH Terms] OR "flour"[All Fields] OR "flours"[All Fields] OR "flour s"[All Fields]) AND ("industrial development"[MeSH Terms] OR ("industrial"[All Fields] AND "development"[All Fields]) OR "industrial development"[All Fields] OR "industrialization"[All Fields] OR "industrialize"[All Fields] OR "industrialized"[All Fields] OR "industrializing"[All Fields] OR "industrially"[All Fields] OR "industrials"[All Fields] OR "industry"[MeSH Terms] OR "industry"[All Fields] OR "industrial"[All Fields] OR "industries"[All Fields] OR "industry s"[All Fields])) OR (("construct s"[All Fields] OR "constructed"[All Fields] OR "constructing"[All Fields] OR "construction"[All Fields] OR "constructions"[All Fields] OR "constructive"[All Fields] OR "constructively"[All Fields] OR "constructs"[All Fields] OR "dna, recombinant"[MeSH Terms] OR ("dna"[All Fields] AND "recombinant"[All Fields]) OR "recombinant dna"[All Fields] OR "construct"[All Fields]) AND ("factories"[All Fields] OR "factory"[All Fields] OR "factory s"[All Fields])) OR (("fuel lond"[Journal] OR "fuel"[All Fields]) AND ("station"[All Fields] OR "station s"[All Fields] OR "stationed"[All Fields] OR "stationing"[All Fields] OR "stations"[All Fields])) OR (("cement s"[All Fields] OR "cementable"[All Fields] OR "cementation"[MeSH Terms] OR "cementation"[All Fields] OR "cementations"[All Fields] OR "cementing"[All Fields] OR "dental cementum"[MeSH Terms] OR ("dental"[All Fields] AND "cementum"[All Fields]) OR "dental cementum"[All Fields] OR "cement"[All Fields] OR "dental cements"[MeSH Terms] OR ("dental"[All Fields] AND "cements"[All Fields]) OR "dental cements"[All Fields] OR "cemented"[All Fields] OR "cements"[All Fields]) AND ("industrial development"[MeSH Terms] OR ("industrial"[All Fields] AND "development"[All Fields]) OR "industrial development"[All Fields] OR "industrialization"[All Fields] OR "industrialize"[All Fields] OR "industrialized"[All Fields] OR "industrializing"[All Fields] OR "industrially"[All Fields] OR "industrials"[All Fields] OR "industry"[MeSH Terms] OR "industry"[All Fields] OR "industrial"[All Fields] OR "industries"[All Fields] OR "industry s"[All Fields])) OR (("gossypium"[MeSH Terms] OR "gossypium"[All Fields] OR "cotton"[All Fields] OR "cottons"[All Fields]) AND "ginning"[All Fields] AND ("industrial development"[MeSH Terms] OR ("industrial"[All Fields] AND "development"[All Fields]) OR "industrial development"[All Fields] OR "industrialization"[All Fields] OR "industrialize"[All Fields] OR "industrialized"[All Fields] OR "industrializing"[All Fields] OR "industrially"[All Fields] OR "industrials"[All Fields] OR "industry"[MeSH Terms] OR "industry"[All Fields] OR "industrial"[All Fields] OR "industries"[All Fields] OR "industry s"[All Fields])) OR (("wood"[MeSH Terms] OR "wood"[All Fields]) AND ("industrial development"[MeSH Terms] OR ("industrial"[All Fields] AND "development"[All Fields]) OR "industrial development"[All Fields] OR "industrialization"[All Fields] OR "industrialize"[All Fields] OR "industrialized"[All Fields] OR "industrializing"[All Fields] OR "industrially"[All Fields] OR "industrials"[All Fields] OR "industry"[MeSH Terms] OR "industry"[All Fields] OR "industrial"[All Fields] OR "industries"[All Fields] OR "industry s"[All Fields])) OR (("metal s"[All Fields] OR "metalate"[All Fields] OR "metalated"[All Fields] OR "metalates"[All Fields] OR "metalating"[All Fields] OR "metalation"[All Fields] OR "metalations"[All Fields] OR "metalative"[All Fields] OR "metalic"[All Fields] OR "metalization"[All Fields] OR "metalized"[All Fields] OR "metallate"[All Fields] OR "metallated"[All Fields] OR "metallates"[All Fields] OR "metallation"[All Fields] OR "metallations"[All Fields] OR "metallic"[All Fields] OR "metallically"[All Fields] OR "metallicities"[All Fields] OR "metallicity"[All Fields] OR "metallics"[All Fields] OR "metallization"[All Fields] OR "metallizations"[All Fields] OR "metallize"[All Fields] OR "metallized"[All Fields] OR "metals"[MeSH Terms] OR "metals"[All Fields] OR "metal"[All Fields]) AND ("industrial development"[MeSH Terms] OR ("industrial"[All Fields] AND "development"[All Fields]) OR "industrial development"[All Fields] OR "industrialization"[All Fields] OR "industrialize"[All Fields] OR "industrialized"[All Fields] OR "industrializing"[All Fields] OR "industrially"[All Fields] OR "industrials"[All Fields] OR "industry"[MeSH Terms] OR "industry"[All Fields] OR "industrial"[All Fields] OR "industries"[All Fields] OR "industry s"[All Fields])) OR ("factor"[All Fields] OR "factor s"[All Fields] OR "factors"[All Fields]) OR ("analysis"[MeSH Subheading] OR "analysis"[All Fields] OR "determination"[All Fields] OR "determinant"[All Fields] OR "determinants"[All Fields] OR "determinate"[All Fields] OR "determinated"[All Fields] OR "determinates"[All Fields] OR "determinating"[All Fields] OR "determinations"[All Fields] OR "determine"[All Fields] OR "determined"[All Fields] OR "determines"[All Fields] OR "determining"[All Fields]) OR ("predictor"[All Fields] OR "predictors"[All Fields]) OR (("factor"[All Fields] OR "factor s"[All Fields] OR "factors"[All Fields]) AND ("associate"[All Fields] OR "associated"[All Fields] OR "associates"[All Fields] OR "associating"[All Fields] OR "association"[MeSH Terms] OR "association"[All Fields] OR "associations"[All Fields])) OR (("associate"[All Fields] OR "associated"[All Fields] OR "associates"[All Fields] OR "associating"[All Fields] OR "association"[MeSH Terms] OR "association"[All Fields] OR "associations"[All Fields]) AND ("factor"[All Fields] OR "factor s"[All Fields] OR "factors"[All Fields])) OR ("risk factors"[MeSH Terms] OR ("risk"[All Fields] AND "factors"[All Fields]) OR "risk factors"[All Fields])) AND ("ethiopia"[MeSH Terms] OR "ethiopia"[All Fields] OR "ethiopia s"[All Fields])

**Translations**

**prevalence:** "epidemiology"[Subheading] OR "epidemiology"[All Fields] OR "prevalence"[All Fields] OR "prevalence"[MeSH Terms] OR "prevalance"[All Fields] OR "prevalences"[All Fields] OR "prevalence's"[All Fields] OR "prevalent"[All Fields] OR "prevalently"[All Fields] OR "prevalents"[All Fields]

**proportion**: "proportion"[All Fields] OR "proportions"[All Fields]

**magnitude:** "magnitude"[All Fields] OR "magnitudes"[All Fields]

**personal protective equipment:** "personal protective equipment"[MeSH Terms] OR ("personal"[All Fields] AND "protective"[All Fields] AND "equipment"[All Fields]) OR "personal protective equipment"[All Fields]

**PPE:** "Polit Philos Econ"[Journal:__jid101738167] OR "ppe"[All Fields]

**personal:** "person's"[All Fields] OR "personable"[All Fields] OR "personableness"[All Fields] OR "personal"[All Fields] OR "personalisation"[All Fields] OR "personalise"[All Fields] OR "personalised"[All Fields] OR "personalising"[All Fields] OR "personality"[MeSH Terms] OR "personality"[All Fields] OR "personalities"[All Fields] OR "personality's"[All Fields] OR "personalization"[All Fields] OR "personalize"[All Fields] OR "personalized"[All Fields] OR "personalizes"[All Fields] OR "personalizing"[All Fields] OR "personally"[All Fields] OR "personals"[All Fields] OR "persons"[MeSH Terms] OR "persons"[All Fields] OR "person"[All Fields]

**safety equipment:** "protective devices"[MeSH Terms] OR ("protective"[All Fields] AND "devices"[All Fields]) OR "protective devices"[All Fields] OR ("safety"[All Fields] AND "equipment"[All Fields]) OR "safety equipment"[All Fields]

**utilization:** "statistics and numerical data"[Subheading] OR ("statistics"[All Fields] AND "numerical"[All Fields] AND "data"[All Fields]) OR "statistics and numerical data"[All Fields] OR "utilization"[All Fields] OR "utilisation"[All Fields] OR "utilisations"[All Fields] OR "utilise"[All Fields] OR "utilised"[All Fields] OR "utilises"[All Fields] OR "utilising"[All Fields] OR "utilities"[All Fields] OR "utility"[All Fields] OR "utilizations"[All Fields] OR "utilize"[All Fields] OR "utilized"[All Fields] OR "utilizer"[All Fields] OR "utilizers"[All Fields] OR "utilizes"[All Fields] OR "utilizing"[All Fields]

**practice:** "practicability"[All Fields] OR "practicable"[All Fields] OR "practical"[All Fields] OR "practicalities"[All Fields] OR "practicality"[All Fields] OR "practically"[All Fields] OR "practicals"[All Fields] OR "practice"[All Fields] OR "practice's"[All Fields] OR "practiced"[All Fields] OR "practices"[All Fields] OR "practicing"[All Fields]

**industry:** "industrial development"[MeSH Terms] OR ("industrial"[All Fields] AND "development"[All Fields]) OR "industrial development"[All Fields] OR "industrialization"[All Fields] OR "industrialize"[All Fields] OR "industrialized"[All Fields] OR "industrializing"[All Fields] OR "industrially"[All Fields] OR "industrials"[All Fields] OR "industry"[MeSH Terms] OR "industry"[All Fields] OR "industrial"[All Fields] OR "industries"[All Fields] OR "industry's"[All Fields]

**factory:** "factories"[All Fields] OR "factory"[All Fields] OR "factory's"[All Fields]

small: "Small"[Journal:__jid101235338] OR "small"[All Fields]

**scale:** "scale's"[All Fields] OR "scaled"[All Fields] OR "scaling"[All Fields] OR "scalings"[All Fields] OR "weights and measures"[MeSH Terms] OR ("weights"[All Fields] AND "measures"[All Fields]) OR "weights and measures"[All Fields] OR "scale"[All Fields] OR "scales"[All Fields]

**industry:** "industrial development"[MeSH Terms] OR ("industrial"[All Fields] AND "development"[All Fields]) OR "industrial development"[All Fields] OR "industrialization"[All Fields] OR "industrialize"[All Fields] OR "industrialized"[All Fields] OR "industrializing"[All Fields] OR "industrially"[All Fields] OR "industrials"[All Fields] OR "industry"[MeSH Terms] OR "industry"[All Fields] OR "industrial"[All Fields] OR "industries"[All Fields] OR "industry's"[All Fields]

**small:** "Small"[Journal:__jid101235338] OR "small"[All Fields]

**scale:** "scale's"[All Fields] OR "scaled"[All Fields] OR "scaling"[All Fields] OR "scalings"[All Fields] OR "weights and measures"[MeSH Terms] OR ("weights"[All Fields] AND "measures"[All Fields]) OR "weights and measures"[All Fields] OR "scale"[All Fields] OR "scales"[All Fields]

**enterprise:** "enterprise"[All Fields] OR "enterprise's"[All Fields] OR "enterprisers"[All Fields] OR "enterprises"[All Fields] OR "enterprising"[All Fields]

**workers:** "occupational groups"[MeSH Terms] OR ("occupational"[All Fields] AND "groups"[All Fields]) OR "occupational groups"[All Fields] OR "worker"[All Fields] OR "workers"[All Fields] OR "worker's"[All Fields]

small: "Small"[Journal:__jid101235338] OR "small"[All Fields]

scale: "scale's"[All Fields] OR "scaled"[All Fields] OR "scaling"[All Fields] OR "scalings"[All Fields] OR "weights and measures"[MeSH Terms] OR ("weights"[All Fields] AND "measures"[All Fields]) OR "weights and measures"[All Fields] OR "scale"[All Fields] OR "scales"[All Fields]

**industry:** "industrial development"[MeSH Terms] OR ("industrial"[All Fields] AND "development"[All Fields]) OR "industrial development"[All Fields] OR "industrialization"[All Fields] OR "industrialize"[All Fields] OR "industrialized"[All Fields] OR "industrializing"[All Fields] OR "industrially"[All Fields] OR "industrials"[All Fields] OR "industry"[MeSH Terms] OR "industry"[All Fields] OR "industrial"[All Fields] OR "industries"[All Fields] OR "industry's"[All Fields]

**workers:** "occupational groups"[MeSH Terms] OR ("occupational"[All Fields] AND "groups"[All Fields]) OR "occupational groups"[All Fields] OR "worker"[All Fields] OR "workers"[All Fields] OR "worker's"[All Fields]

**large:** "large"[All Fields] OR "largely"[All Fields] OR "larges"[All Fields]

**scale:** "scale's"[All Fields] OR "scaled"[All Fields] OR "scaling"[All Fields] OR "scalings"[All Fields] OR "weights and measures"[MeSH Terms] OR ("weights"[All Fields] AND "measures"[All Fields]) OR "weights and measures"[All Fields] OR "scale"[All Fields] OR "scales"[All Fields]

**industry:** "industrial development"[MeSH Terms] OR ("industrial"[All Fields] AND "development"[All Fields]) OR "industrial development"[All Fields] OR "industrialization"[All Fields] OR "industrialize"[All Fields] OR "industrialized"[All Fields] OR "industrializing"[All Fields] OR "industrially"[All Fields] OR "industrials"[All Fields] OR "industry"[MeSH Terms] OR "industry"[All Fields] OR "industrial"[All Fields] OR "industries"[All Fields] OR "industry's"[All Fields]

**large:** "large"[All Fields] OR "largely"[All Fields] OR "larges"[All Fields]

**scale**: "scale's"[All Fields] OR "scaled"[All Fields] OR "scaling"[All Fields] OR "scalings"[All Fields] OR "weights and measures"[MeSH Terms] OR ("weights"[All Fields] AND "measures"[All Fields]) OR "weights and measures"[All Fields] OR "scale"[All Fields] OR "scales"[All Fields]

industry: "industrial development"[MeSH Terms] OR ("industrial"[All Fields] AND "development"[All Fields]) OR "industrial development"[All Fields] OR "industrialization"[All Fields] OR "industrialize"[All Fields] OR "industrialized"[All Fields] OR "industrializing"[All Fields] OR "industrially"[All Fields] OR "industrials"[All Fields] OR "industry"[MeSH Terms] OR "industry"[All Fields] OR "industrial"[All Fields] OR "industries"[All Fields] OR "industry's"[All Fields]

**workers:** "occupational groups"[MeSH Terms] OR ("occupational"[All Fields] AND "groups"[All Fields]) OR "occupational groups"[All Fields] OR "worker"[All Fields] OR "workers"[All Fields] OR "worker's"[All Fields]

**textile industry:** "textile industry"[MeSH Terms] OR ("textile"[All Fields] AND "industry"[All Fields]) OR "textile industry"[All Fields]

**tannery:** "tanneries"[All Fields] OR "tannery"[All Fields]

**industry:** "industrial development"[MeSH Terms] OR ("industrial"[All Fields] AND "development"[All Fields]) OR "industrial development"[All Fields] OR "industrialization"[All Fields] OR "industrialize"[All Fields] OR "industrialized"[All Fields] OR "industrializing"[All Fields] OR "industrially"[All Fields] OR "industrials"[All Fields] OR "industry"[MeSH Terms] OR "industry"[All Fields] OR "industrial"[All Fields] OR "industries"[All Fields] OR "industry's"[All Fields]

**flour:** "flour"[MeSH Terms] OR "flour"[All Fields] OR "flours"[All Fields] OR "flour's"[All Fields]

**industry:** "industrial development"[MeSH Terms] OR ("industrial"[All Fields] AND "development"[All Fields]) OR "industrial development"[All Fields] OR "industrialization"[All Fields] OR "industrialize"[All Fields] OR "industrialized"[All Fields] OR "industrializing"[All Fields] OR "industrially"[All Fields] OR "industrials"[All Fields] OR "industry"[MeSH Terms] OR "industry"[All Fields] OR "industrial"[All Fields] OR "industries"[All Fields] OR "industry's"[All Fields]

**construction:** "construct's"[All Fields] OR "constructed"[All Fields] OR "constructing"[All Fields] OR "construction"[All Fields] OR "construction's"[All Fields] OR "constructions"[All Fields] OR "constructive"[All Fields] OR "constructively"[All Fields] OR "constructs"[All Fields] OR "dna, recombinant"[MeSH Terms] OR ("dna"[All Fields] AND "recombinant"[All Fields]) OR "recombinant dna"[All Fields] OR "construct"[All Fields]

factory: "factories"[All Fields] OR "factory"[All Fields] OR "factory's"[All Fields]

**Fuel:** "Fuel (Lond)"[Journal:__jid101556828] OR "fuel"[All Fields]

station: "station"[All Fields] OR "station's"[All Fields] OR "stationed"[All Fields] OR "stationing"[All Fields] OR "stations"[All Fields]

**cement:** "cement's"[All Fields] OR "cementable"[All Fields] OR "cementation"[MeSH Terms] OR "cementation"[All Fields] OR "cementations"[All Fields] OR "cementing"[All Fields] OR "dental cementum"[MeSH Terms] OR ("dental"[All Fields] AND "cementum"[All Fields]) OR "dental cementum"[All Fields] OR "cement"[All Fields] OR "dental cements"[MeSH Terms] OR ("dental"[All Fields] AND "cements"[All Fields]) OR "dental cements"[All Fields] OR "cemented"[All Fields] OR "cements"[All Fields]

**industry:** "industrial development"[MeSH Terms] OR ("industrial"[All Fields] AND "development"[All Fields]) OR "industrial development"[All Fields] OR "industrialization"[All Fields] OR "industrialize"[All Fields] OR "industrialized"[All Fields] OR "industrializing"[All Fields] OR "industrially"[All Fields] OR "industrials"[All Fields] OR "industry"[MeSH Terms] OR "industry"[All Fields] OR "industrial"[All Fields] OR "industries"[All Fields] OR "industry's"[All Fields]

cotton: "gossypium"[MeSH Terms] OR "gossypium"[All Fields] OR "cotton"[All Fields] OR "cottons"[All Fields]

industry: "industrial development"[MeSH Terms] OR ("industrial"[All Fields] AND "development"[All Fields]) OR "industrial development"[All Fields] OR "industrialization"[All Fields] OR "industrialize"[All Fields] OR "industrialized"[All Fields] OR "industrializing"[All Fields] OR "industrially"[All Fields] OR "industrials"[All Fields] OR "industry"[MeSH Terms] OR "industry"[All Fields] OR "industrial"[All Fields] OR "industries"[All Fields] OR "industry's"[All Fields]

wood: "wood"[MeSH Terms] OR "wood"[All Fields]

**industry:** "industrial development"[MeSH Terms] OR ("industrial"[All Fields] AND "development"[All Fields]) OR "industrial development"[All Fields] OR "industrialization"[All Fields] OR "industrialize"[All Fields] OR "industrialized"[All Fields] OR "industrializing"[All Fields] OR "industrially"[All Fields] OR "industrials"[All Fields] OR "industry"[MeSH Terms] OR "industry"[All Fields] OR "industrial"[All Fields] OR "industries"[All Fields] OR "industry's"[All Fields]

**metal:** "metal's"[All Fields] OR "metalate"[All Fields] OR "metalated"[All Fields] OR "metalates"[All Fields] OR "metalating"[All Fields] OR "metalation"[All Fields] OR "metalations"[All Fields] OR "metalative"[All Fields] OR "metalic"[All Fields] OR "metalization"[All Fields] OR "metalized"[All Fields] OR "metallate"[All Fields] OR "metallated"[All Fields] OR "metallates"[All Fields] OR "metallation"[All Fields] OR "metallations"[All Fields] OR "metallic"[All Fields] OR "metallically"[All Fields] OR "metallicities"[All Fields] OR "metallicity"[All Fields] OR "metallics"[All Fields] OR "metallization"[All Fields] OR "metallizations"[All Fields] OR "metallize"[All Fields] OR "metallized"[All Fields] OR "metals"[MeSH Terms] OR "metals"[All Fields] OR "metal"[All Fields]

industry: "industrial development"[MeSH Terms] OR ("industrial"[All Fields] AND "development"[All Fields]) OR "industrial development"[All Fields] OR "industrialization"[All Fields] OR "industrialize"[All Fields] OR "industrialized"[All Fields] OR "industrializing"[All Fields] OR "industrially"[All Fields] OR "industrials"[All Fields] OR "industry"[MeSH Terms] OR "industry"[All Fields] OR "industrial"[All Fields] OR "industries"[All Fields] OR "industry's"[All Fields]

**factors:** "factor"[All Fields] OR "factor's"[All Fields] OR "factors"[All Fields]

**determinants**: "analysis"[Subheading] OR "analysis"[All Fields] OR "determination"[All Fields] OR "determinant"[All Fields] OR "determinants"[All Fields] OR "determinate"[All Fields] OR "determinated"[All Fields] OR "determinates"[All Fields] OR "determinating"[All Fields] OR "determinations"[All Fields] OR "determine"[All Fields] OR "determined"[All Fields] OR "determines"[All Fields] OR "determining"[All Fields]

**predictors:** "predictor"[All Fields] OR "predictors"[All Fields]

**factors**: "factor"[All Fields] OR "factor's"[All Fields] OR "factors"[All Fields]

**associated:** "associate"[All Fields] OR "associated"[All Fields] OR "associates"[All Fields] OR "associating"[All Fields] OR "association"[MeSH Terms] OR "association"[All Fields] OR "associations"[All Fields]

**associated:** "associate"[All Fields] OR "associated"[All Fields] OR "associates"[All Fields] OR "associating"[All Fields] OR "association"[MeSH Terms] OR "association"[All Fields] OR "associations"[All Fields]

**factors:** "factor"[All Fields] OR "factor's"[All Fields] OR "factors"[All Fields]

**risk factors**: "risk factors"[MeSH Terms] OR ("risk"[All Fields] AND "factors"[All Fields]) OR "risk factors"[All Fields]

**Ethiopia:** "ethiopia"[MeSH Terms] OR "ethiopia"[All Fields] OR "ethiopia's"[All Fields]
